# Efficacy of sirolimus in children with lymphatic malformations of the head and neck

**DOI:** 10.1007/s00405-022-07378-8

**Published:** 2022-05-08

**Authors:** S. Wiegand, A. Dietz, G. Wichmann

**Affiliations:** Department of Otolaryngology, Head and Neck Surgery, University of Leipzig, Liebigstr. 12, 04103 Leipzig, Germany

**Keywords:** Lymphatic malformation, Children, Sirolimus, Rapamycin

## Abstract

**Purpose:**

Children with extensive lymphatic malformations of the head and neck often suffer from functional impairment and aesthetic deformity which significantly affect the quality of life and may be life-threatening. Treatment with sirolimus has the potential to improve symptoms and downsize lymphatic malformations. This systematic review summarizes the current information about sirolimus treatment of lymphatic malformations of the head and neck in children, its efficacy and side effects.

**Methods:**

A systematic search of the literature regarding studies on sirolimus treatment of children with lymphatic malformations of the head and neck was performed in PubMed, Embase, and Google Scholar up to July 2021 with the search terms “lymphatic malformation”, “lymphangioma”, “cystic hygroma”, “low-flow malformation”, “sirolimus”, “rapamycin”, “mTOR inhibitor” and “children”.

**Results:**

In all, 28 studies including 105 children from newborn to 17 years treated with sirolimus for lymphatic malformations of the head and neck were analyzed. The most frequent initial dose was 0.8 mg/m^2^ per dose, twice daily at 12-h interval. The target blood level differed between studies, 10–15 ng/mL and 5–15 ng/mL were most often used. More than 91% of the children responded to sirolimus treatment which lasts from 6 months to 4 years. Typical side effects were hyperlipidemia, neutropenia and infections.

**Methods:**

Sirolimus could be an effective treatment for children with large complicated lymphatic malformations of the head and neck. As not all patients will benefit from treatment, the decision to treat sirolimus should be made by a multidisciplinary team.

## Introduction

Lymphatic malformations (LM) are congenital developmental anomalies of the lymphatic system with an estimated incidence of 1 in 2000 live births [1]. Depending on the size of the cysts LM are categorized into macrocystic (> 1 cm), microcystic (< 1 cm), or mixed lesions. LM are usually diagnosed at birth or in the first two years of life, whereas large LM can also be diagnosed prenatally, and some LM have delayed presentation in adulthood. The precise pathogenesis of LM is still unknown but in some patients they are associated with syndromic disorders like Proteus and CLOVES syndrome [2, 3]. LM most frequently occur in the head and neck region, but they can also be found in other parts of the body, especially in lymphatic-rich areas. Symptoms depend on the size and location of LM. In the head and neck area, symptoms can range from minimal swelling to life-threatening airway obstruction, impaired oral feeding, macroglossia, overgrowth of the mandible, loss of vision, pain and aesthetic disfigurement [4, 5]. Relapsing periods of infection, trauma or intracystic hemorrhage can lead to acute enlargement while LM usually grows proportional to the patients’ body growth.

The management of LM often necessitates a multidisciplinary approach and an individualized treatment depending on symptoms, presence of functional compromise, size and location. Surgery is a mainstay of treatment, especially in microcystic and mixed LM. Sclerotherapy is particularly effective in macrocystic LM and as an adjunctive therapy used in combination with surgery. There were different possible sclerosants, however, picibanil (OK-432) and doxycycline are most often used [6]. Laser therapy to vaporize the tissue and seal superficial lymphatic channels is mainly effective in mucosal LM. In absence of functional deficits and symptoms watch-and-wait may also may also be an option. Pharmacologic treatment with different drugs has also been considered for the treatment of large LM, especially in patients who were refractory to conventional treatments. The most promising systemic treatment seems to be sirolimus that was demonstrated to be useful in the therapy of certain diseases and has been shown to inhibit lymphangiogenesis [7–10].

Sirolimus is also known as rapamycin, as it was initially discovered in a soil sample of Rapa Nui (Easter Island). It is a natural macrolide derived from the soil bacteria *Streptomyces hygrosopicus*. Sirolimus is a specific inhibitor of the mammalian target of rapamycin (mTOR), a serine/threonine kinase that is a key factor in the regulation of angiogenesis, cell growth and proliferation. Sirolimus was approved by the FDA in 1999 as an oral immunosuppressive for use in renal transplantation and in 2015 as the first drug to treat lymphangioleiomyomatosis, a rare disease that is characterized by progressive, cystic lung disease, angiomyolipoma and lymphangioleiomyomas and predominately affects young women. Owing to its antiangiogenic and antiproliferative properties, sirolimus treatment was used to treat large LM. In a previous systematic review, it could be demonstrated that treatment with sirolimus has the potential to improve symptoms and downsize LM of different body areas [11]. The present review aims to analyze the available information about efficacy and side effects in children with LM of the head and neck.

## Material and methods

A systematic search of the literature was performed in PubMed, Embase, and Google Scholar. The search terms “lymphatic malformation”, “lymphangioma”, “cystic hygroma”, “low-flow mal-formation”, “sirolimus”, “rapamycin”, “mTOR inhibitor” and “children” were used in “AND” and “OR” combinations. The search was limited to articles published until July 2021. All studies focusing on systemic sirolimus therapy for head and neck LM in children (< 18 years) that were presented in English, Spanish, French and German languages were eligible for inclusion. Studies reporting non-original data (systematic reviews, meta-analyses, narrative reviews, commentaries, correspondence, letters), studies addressing both adults and children or LM of locations inside and outside the head and neck and that did not report data from the head and neck or children separately, duplicate publications, as well as reports with insufficient information were excluded. Reports on topical treatment were also excluded. In addition to the electronic database search, the reference sections of previous systematic reviews and the included articles were checked for further potentially relevant articles. The literature search and the evaluation of inclusion criteria were performed by the first author with uncertainties resolved through consultation among all the authors. The initial screening was based on the titles and/or abstracts. Next, the hard copies of the potentially eligible publications were examined to assess whether they met the inclusion criteria. Reports on the generalized lymphatic anomaly, Gorham-Stout disease, lymphangiomatosis, lymphangiectasia, and chronic lymphedema as well as studies on patients with venolymphatic and capillary-lymphatico-venous malformations were not included in this review.

For each selected report, the following variables were considered: year and country of publication, study design, number of cases described, sex and age of the patients, location of LM, treatment (including dosage and target serum level), treatment duration, outcome, adverse events and follow-up.

## Results

### Clinical characteristics of the patients

In all, 28 case series and case reports and two prospective trials [31, 36] including 105 children treated with sirolimus for LM of the head and neck, were included in this review (Table 1) [12–39]. Due to the inclusion criteria, the age of the patients ranged from newborn to 17 years. The male to female ratio was 1:1.21, but the sex was not reported for all patients. In 51 patients (48.6%), pretreatment using other modalities was reported to be insufficient without adequate response. The most common pretreatments were sclerotherapy (*n* = 30), surgery (*n* = 7) and the combination of surgery and sclerotherapy (*n* = 7). Other pretreatments were intralesional triamcinolone, steroids combined with propranolol, sildenafil and propranolol, prednisone, laser therapy as well as surgery combined with laser therapy.

**Table 1:** Studies concerning sirolimus therapy of lymphatic malformations of the head and neck in children

Author	Year	No. of patients	Localization	Age	Sex	Starting dose	Target blood level (ng/mL)	Parallel treatment	Result	Therapy duration	Adverse events
Tschauner [12]	2013	4	Neck	6 Mo-4 Y	F	NR	NR	No	4 PR (49–75%)	NR	NR
Akyüz [13]	2014	1	Tongue	10 Y	M	0.8 mg/m^2^ per dose twice daily	NR	No	PR (70%)	12 Mo	NR
Margolin [14]	2014	1	Neck, tongue, mediastinum, floor of mouth	17 Mo	F	NR	10–15	No	PR	12 Mo (ongoing)	NR
Alemi [15]	2015	2	Larynx, neck, mediastinum face, neck, chest wall, larynx, mediastinum, oropharynx	4 Mo 1 Mo	M NR	NR	NR	Laser surgery No	PR PR	2y, 8 mo discontinued, 5 mo (onging) 11 mo (ongoing)	No
Lackner [16]	2015	1	Orbit	9 Y	M	0.05 mg/kg twice daily perorally	5–15	No	PR	6 Mo	Mild reversible leukopenia
Yesil [17]	2015	1	Tongue	1 Y	F	0.8 mg/m^2^ per dose twice daily	10–15	No	PR (90%)	9 Mo	Hypertriglyceridemia
Azouz [18]	2016	1	Neck, cheek, tongue	1 Mo	M	0.07 mg/kg given twice daily	NR	No	PR	15 mo	NR
Altawil [19]	2017	2	Neck Parotid/neck	20 Mo 12 Y	F M	0.8 mg/m^2^ per dose twice daily	10–13	No: 1 sclerotherapy: 1	2 PR	6 weeks (ongoing) 8 weeks (ongoing)	Neutropenia (2)
Amodeo [20]	2017	3	Face, neck,upper mediastinum	2,5 Mo, 16 Mo, 23 Mo	2 M 1 F	0.4–0.8 mg/m2 per dose twice daily	10–15	No: 2 surgery: 1	2 PR	1 > 6 Months 1 > 2 Months NR	Dyslipidemia (1)
Escoda [21]	2017	1	Neck	Newborn	M	0.8 mg/m^2^ per dose twice daily	4–8	No	PR	2 Mo (ongoing)	Hypertriglyceridaemia, hypercholesterolaemia, nausea, vomiting
Thirion [22]	2017	2	Retroorbital cervical	2 Mo 12 Years	M	NR	NR	No	2 PR	10 Mo (ongoing) 2y (ongoing)	No
Strychowsky [23]	2018	15	Cervicofacial	2 Mo–17 Y	NR	0.8 mg/m^2^ per dose twice daily	10–15	No	15 PR	0,64Y-3,42Y 4 patients ongoing (1,55–2,98 Y)	Cellulitis (6) Nausea/emesis (3) Eczema (6) Mouth sores (9) Transaminitis (5), elevated cholesterol and troglycerides (4) Neutropenia (3) Rash (1) Acne (1) Diarrhea (1) Headache (3) Alopecia (1) Irregular menstrual Bleeding (1)
Hammer [24]	2018	3	Cervicofacial, larynx parotid, tongue, retroorbital	3y, 11 y, 16y,	2 F 1 M	0.8 mg/m^2^ per dose twice daily	10–15	No	2 PR 1 SD	13 Mo, 34 Mo 28 Mo ongoing	Several, not only reported for LM
Triana [25]	2019	6	FACE, NECK, TONGUE, SUBMANDIBULAR REGION	Median 15D (1–32 D)	3 F, 3 M	0.8 mg/m^2^ per dose twice daily	4–12	No	1 CR 5 PR	3–46 Mo	Elevation of gamma-glutamyl transferase (1) and triglycerides (1) lymphangitis (3), intralesional bleeding (2)
Curry [26]	2019	1	Neck, upper chest	22 D	F	0.8 mg/m^2^ per dose twice daily	10–15	No	PR	8 Mo, ongoing	Cellulitis
Ghariani Fetoui [27]	2019	1	Tongue	2 Y	F	0.4–0.8 mg/m^2^/d in two divided doses	10–12	No	PR	6 Mo	Bacterial pneumonia
Gimenez-Aleixandre [28]	2019	3	Neck, cervicofacial, retrobulbar	2 Mo 4 Y 13 Y	f	0.8 mg/m^2^ per dose twice daily	5–15	No	1 PR	10mo ongoing (1) 1,5 y (2)	Hypertransaminasemia (1) hypercholesterinemia (1)
Gonzalez-Hermosa [29]	2019	1	Cervical	19 D	m	0.8 mg/m^2^ per day	NR	No	PR after 12 months	16 Mo	No
Meurisse [30]	2019	2	Neck	21 D 9 weeks	1 m, 1 NR	0.08 mg/kg/day	3,5–6 4–12	Sclero-therapy with polidocanol or ethanol gel	PR (70% and 80%)	8–16 Mo	No
Ozeki [31]	2019	5	Neck, tongue Orbit, mediastinal	2 weeks, 10 mo, 1 y, 2 y, 11 y	2 m, 3 f	1-2 mg once a day	5–15	No	4 PR 1 SD	6–18 Mo	Upper respiratory infection (1), cellulitis (1), stomatitis (1)
Pandey [32]	2019	16	Tongue	0–12 y	NR	0.8 mg/day in three doses	NR	No	14 PR (9 > 75%, 5 > 50%) 2 SD	NR	NR
Sandbank [33]	2019	3	Cervicofacial, tongue	NR	NR	0.6 mg/m^2^ per dose twice daily	5–12	No	3 PR	NR	Hyperkaliaemia, thrombocytosis, increased AST, hypercholesterinaemia
Gomez-Sanchez [34]	2020	3	Cervicofacial, supraglottic and pharyngeal parotid	3 Y, 8 Y, 9 Y	M	0.8 mg/m^2^ per dose twice daily	5–15	No	PR: 3	4 Y ongoing 4 Y ongoing 14 Mo ongiong	Slight cholesterol increase (1), oral enanthema (1)
Honnorat [35]	2020	1	Cervicofacial	1 D	F	0.1 mg per kg per day	12–20	Sclero-therapy with doxycycline and bleomycin, laser therapy	PR	9 Mo ongoing	Mild dyslipidemia, several infections
Zhang [36]	2021	16	Neck	16 D–171 Mo	NR	0.8 mg/m^2^ per dose twice daily 0.5 mg/m^2^ per dose daily in neonates	4–13	No	PR: 14	NR	Oral mucositis, hypercholesterolemia, upper respiratory infections, hepatic dysfunction, dizziness, cystic hemorrhage
Chouchene [37]	2021	1	Tongue	2 Y	F	1.5 mg/kg/day in two divided daily doses	NR	No	PR	6 Mo	No
Harbers [38]	2021	1	Tongue	1 Y	F	NR	4–10	Surgery	PR	6 Mo, interruption, restart for 15 mo, surgery, restart, in all 41 mo, ongoing	Intermittent aphthous stomatitis, upper airway infections, tonsillitis, elevation of triglycerides
Wu [39]	2021	8	Head and neck	2 Mo-3 Y	5 F, 3 M	0.8 mg/m^2^, twice-a-day	10–15	–	At the final observation (12 months) PR: 40% (3), 60% (3), 75% (1), 10% (1)	1.11–1.65y, ongoing	Mouth sores (6), eczema (1), gastrointestinal reaction (2), dyslipidemia (1), neutropenia (1) One patient interrupted sirolimus therapy for recurrent fever caused by upper respiratory tract infection

Abbreviations: *y* year, *mo* months, *d* days, *m* male, *f* female, *NR* not reported, *NE* not evaluable, *PR* partial response, *PD* progressive disease

### Treatment with sirolimus

In most studies, sirolimus was administered orally at an initial dosage of 0.8 mg/m^2^ per dose, twice daily at 12-h intervals [13, 17, 19, 21, 23–26, 28, 34, 36, 39]. In other studies, this was modified to 0.8 mg/m^2^ per day [29], 0.8 mg per day in three doses [32], or 0.08 mg/kg per day [30]. Zhang reported to use a reduced starting dose in neonates (0.5 mg/m^2^). Other starting doses were 1–2 mg per day [31], 0.1 mg/kg per day [35], 0.05 mg/kg twice daily [16], 0.6 mg/m^2^ per twice daily dose [33], 0.07 mg/kg twice daily [18], 0.4–0.8 mg/m^2^ twice daily [20] or in two fractions [27], and 1.5 mg/kg/day administered in two divided daily doses [37]. In five publications, the initial dose of sirolimus was not reported [12, 14, 15, 22, 38]. After the beginning of treatment with sirolimus the dose was then subsequently adjusted to reach the planned target blood level.

The target blood level of sirolimus was ≤ 20 ng/mL in all studies but slightly differed. The target blood level was 10–15 ng/mL in seven studies [14, 17, 20, 23, 24, 26, 39], 5–15 ng/mL in four studies [16, 28, 31, 34] and 4–12 ng/mL in two studies [25, 30]. In one study each, 3.5–6 ng/mL [30], 4–8 ng/mL [21], 4–10 ng/mL [38], 4–13 ng/mL [36], 5–12 ng/mL [33], 10–12 ng/mL [27], 10–13 ng/mL [19] and 12–20 ng/mL [35] were the target concentrations. Eight studies did not report the target blood level of sirolimus [12, 13, 15, 18, 22, 29, 32, 37]. Not in all studies the planned target trough level for sirolimus was achieved [14, 20]. For example, Margolin et al. [14] reported that the sirolimus level was often < 2 ng/mL. Despite difficulties with maintaining the desired sirolimus levels, there was a significant reduction in LM size in the presented case [14].

The time-to-response was not reported in all studies and seem to be variable. For example, Azouz et al. reported a reduction of 70–80% of the LM after 10 days of treatment [18]. Akyüz et al. [13] and Yesil et al. [17] reported a decrease in size of 60% and 70% after 3 months, respectively. Gomez-Sanchez et al. [34] reported a clinical response time from 3.5 to 9 months, Amodeo et al. [20] reported about a response after two months of treatment, while Gonzalez-Hermosa et al. [29] reported 13 months.

In 24 studies, data on the duration of treatment were presented [13–31, 34, 35]. The duration of sirolimus treatment ranged from 6 months to 4 years. In many patients, sirolimus treatment was ongoing at the time of the report.

Parallel treatments additionally to sirolimus were performed in seven cases (6.7%) [15, 19, 20, 30, 35, 38]. In one patient sirolimus was combined with sclerotherapy and laser therapy [35], in two patients with surgery [20, 38], in one patient with laser surgery [15], and in three patients with sclerotherapy [19, 30].

### Treatment efficacy

In all, 96 children (91.4%) responded to sirolimus treatment, 95 patients had a partial response, in one neonate a complete response was reported [25]. This neonate with a large macrocystic cervicofacial LM was treated with sirolimus from the 15^th^ day of live for twelve months. Due to a total involution of the LM at 12 months of life treatment was stopped. There was no recurrence one year later.

In many trials, the volume reduction was not quantified. Terms like “marked improvement”, “significant volume reduction” or “significant decrease” were used. For this reason, a comparison of the results is only possible to a limited extent. In those studies, were the percentage of volume reduction was reported, the reductions were between 49 and 90% [12, 13, 17, 30, 32]. Independent of the decrease of LM, Hammer et al. reported an improvement in pain in all patients [24].

### Side effects

Twenty-three studies commented on possible adverse events associated with and probably being a result of sirolimus therapy [15–17, 19–31, 33–39]. In five studies no patients experienced side effects [15, 22, 29, 30, 37]. In the other 18 studies, different adverse effects were reported [16, 17, 19–21, 23–28, 31, 33–36, 38, 39], the most common were hyperlipidemia, neutropenia and infections such as cellulitis. There was no reporting of adverse events graded according to Common Terminology Criteria for Adverse Events (CTCAE). In three studies [23, 27, 36] sirolimus was interrupted or stopped in some cases due to adverse events. For example, Strychowsky et al. described two patients who interrupted sirolimus therapy for adverse events, in one case intravenous antibiotics were given, the other 13-year-old patient developed alopecia but chose to resume sirolimus 4 months after stopping [23]. Zhang et al. reported that sirolimus was intermittently interrupted due to drug side effects in some cases. The adverse events did not correlate with the blood level of sirolimus [36].

In 46 patients (43.8%) from five studies [22, 23, 31, 32, 34] a prophylactic administration of sulfamethoxazole-trimethoprim for prevention of *Pneumocystis pneumonia* infection was performed. Occurrence of bacterial pneumonia during sirolimus therapy was only reported in one of all investigated cases [27], the causative bacterium was not reported. The sirolimus therapy was withheld in this case, and the girl was treated with a 2-week course of imipenem [27].

## Discussion

Although it is an off-label use, sirolimus has been used for the treatment of vascular anomalies for more than a decade [40, 41] and there is a growing evidence on the effective role of sirolimus in treating LM. In the presented analysis, 90% of the children with LM of the head and neck responded to sirolimus. Besides volume reduction, pain relief and mucosal and skin changes were described. Despite the availability of molecular data demonstrating the impact of sirolimus on various intracellular signaling pathways, the mechanism of action of sirolimus in LM is—also due to the limited understanding of the biology and genetics of LM—not completely understood. The response to sirolimus seems to depend on the microvessel density within the LM suggesting an interference with angiogenetic and lymphogenetic growth factor signaling pathways. Pandey et al. [32] demonstrated that mean lymphatic microvessel density, which was calculated immunohistochemically using the monoclonal antibody D2-40 as the lymphatic endothelial marker, was significantly different in good responders, partial and non-responders to sirolimus. Therefore, they concluded that lymphangiogenesis is a valuable predictive biomarker for the therapeutic response to sirolimus in children with LM [32]. Hori et al. [42] analyzed the activation of the mTOR pathway in a subset of lymphatic anomalies in vivo and demonstrated that normal lymphatic vessels expressed neither mTOR nor its phosphorylated form p‐mTOR. In contrast to normal lymphatic vessels, endothelial cells of LM, kaposiform lymphangiomatosis and kaposiform hemangioendothelioma expressed mTOR. An activation of mTOR was seen in kaposiform lymphangiomatosis and kaposiform hemangioendothelioma but not in LM, whereas the activation of the ribosomal protein S6 kinase 1 (S6K1, an indicator of activated mTOR signaling), and the eukaryotic translation initiation factor 4E‐binding protein (4EBP1) which mediate an increase in protein synthesis and cell growth in lymphangiogenesis was seen in all cases of kaposiform lymphangiomatosis and kaposiform hemangioendothelioma and 40% of LM. Although sirolimus is effective in LM, most of the LM were negative for its phosphorylated form p‐mTOR. Hori et al. explained this fact by possible dual inhibition of mTOR and vascular endothelial growth factor (VEGF) pathway by sirolimus in lymphatic anomalies which both play a vital role in lymphangiogenesis [42, 43]. They assumed that sirolimus may inhibit lymphangiogenesis at least in part by downregulating VEGF expression in p‐mTOR negative cases [42] as it was previously demonstrated that sirolimus treatment also inhibited the expression of VEGFR‐3 and VEGF‐C, the potent growth factors for lymphatic endothelial cells [44, 45]. However, prospective randomized trials concerning this issue are lacking.

Strychowsky et al. [23] reported that macrocystic LM may respond better than mixed or microcystic LM, and younger patients may respond better than older patients to sirolimus. It could be demonstrated that sirolimus can be well-tolerated even in preterm children [35]. It is unclear if there is variable efficacy of sirolimus treatment during different developmental time periods. Pre-treated patients seem to respond less well [23] but there may be synergistic effects with other treatments like sclerotherapy or surgery [18].

The duration of sirolimus treatment differed between the analyzed studies. Most children were treated for several months and sirolimus was not withdrawn at last follow-up. Freixo et al. [46] demonstrated for vascular anomalies that 75% of the first clinical response was reported in less than 21 days. In this review, the time to (reported) clinical response varied between 10 days [18] and 13 months [29]. However, the time to reach the optimum treatment effect remains unclear.

The ability to measure serum levels of sirolimus makes sirolimus treatment controllable to a certain extent. However, a serum level for an effective and safe treatment has not been defined so far. The optimal doses and corresponding plasma levels for each patient to achieve response are still not defined. The target levels, however, were most frequently 5 to 15 ng/mL. Most authors maintained sirolimus at the standard dose. Some authors recommend to reduce the dose after response to sirolimus reached a plateau for some months [23, 25] to achieve the minimal effective dose for each patient to remain asymptomatic. Also, after discontinuation of the drug, some patients had stable disease.

The off-label use of sirolimus in children with LM requires a thoughtful risk–benefit analysis and careful follow-up. Children treated with sirolimus for LM may be at a significant risk for severe adverse events and therefore should be monitored carefully. A retrospective chart review in different European centers demonstrated that most serious adverse events were observed in the first year of sirolimus therapy; but serious adverse events can also occur after a longer treatment period [41]. The occurrence of serious adverse events during sirolimus treatment did not seem to be dependent on the sirolimus blood level [41].

One infrequent but severe side effect is *Pneumocystis Jirovecii* pneumonia, which was reported in patients who underwent solid organ transplants and two patients with vascular anomalies treated with sirolimus [47–49]. Therefore, prophylaxis with sulfamethoxazole-trimethoprime is recommended by some authors [22, 23, 31, 32, 34, 41]. In the presented patient group, nearly half of the children received prophylactic antibiotic treatment with sulfamethoxazole-trimethoprime [22, 23, 31, 32, 34]. However, anti-infectious prophylactic measures were not standardized as data are lacking. Occurrence of bacterial pneumonia during sirolimus therapy was only reported in one of the presented patients [27], however, the causative bacterium was not reported. It is suspected that higher levels of sirolimus or concomitant medications such as corticosteroids may be a risk factor for *Pneumocystis* pneumonia [25] and therefore selected patients may benefit from *Pneumocystis* prophylaxis while on sirolimus.

This systematic review is not without limitations. Inherent to the design we have to deal with missing data which could have impacted the results. We observed a lack in standardization in reporting and its comprehensiveness as relevant details often were missing. In many studies, outcome measures were qualitative and not quantitative, moreover, the outcome was reported at different timepoints. There were variations in the age of the patients and treatment phases (with/without prior treatment), clinical management and treatment durations. The methodological quality of the included studies is low and there was a heterogeneity regarding drug dosage, definition of response, measurement of response and measurement of toxicity. Finally, there may be publication bias with respect to centers publishing good outcomes.

## Conclusion

Sirolimus could be an effective treatment in children with large complicated LM of the head and neck. Until now, data are lacking to define the optimal place of sirolimus in the management strategy. Patients and their parents should be well educated on potential adverse effects and the decision for sirolimus treatment should be made by a multidisciplinary team. However, not all patients benefit from treatment. Therefore, it will be important to define factors predicting response to sirolimus treatment and patients who will benefit from sirolimus. Possibly, the expression of key components of the mTOR signaling pathway (S6K1) and VEGF C and VEGFR3 can serve as a biomarker predicting treatment success. There is a need for further clinical trials in children with head and neck LM establishing the perfect timepoint for initiation and the duration of sirolimus treatment and optimal doses, which will likely depend on the phenotype and extension of the LM, the age of the child and the symptoms.
